# Taxonomic dependency of beta diversity for bacteria, archaea, and fungi in a semi-arid lake

**DOI:** 10.3389/fmicb.2022.998496

**Published:** 2022-11-03

**Authors:** Haijun Yuan, Weizhen Zhang, Huaqun Yin, Runyu Zhang, Jianjun Wang

**Affiliations:** ^1^State Key Laboratory of Lake Science and Environment, Nanjing Institute of Geography and Limnology, Chinese Academy of Sciences, Nanjing, China; ^2^State Key Laboratory of Environmental Geochemistry, Institute of Geochemistry, Chinese Academy of Sciences, Guiyang, China; ^3^University of Chinese Academy of Sciences, Beijing, China; ^4^School of Minerals Processing and Bioengineering, Central South University, Changsha, China; ^5^Key Laboratory of Biometallurgy of Ministry of Education, Central South University, Changsha, China

**Keywords:** beta diversity partitioning, microbial diversity, local contributions to beta diversity, water depth, semi-arid lake

## Abstract

Microbial beta diversity has been recently studied along the water depth in aquatic ecosystems, however its turnover and nestedness components remain elusive especially for multiple taxonomic groups. Based on the beta diversity partitioning developed by Baselga and Local Contributions to Beta Diversity (LCBD) partitioning by Legendre, we examined the water-depth variations in beta diversity components of bacteria, archaea and fungi in surface sediments of Hulun Lake, a semi-arid lake in northern China, and further explored the relative importance of environmental drivers underlying their patterns. We found that the relative abundances of *Proteobacteria*, *Chloroflexi*, *Euryarchaeota*, and *Rozellomycota* increased toward deep water, while *Acidobacteria*, *Parvarchaeota*, and *Chytridiomycota* decreased. For bacteria and archaea, there were significant (*p* < 0.05) decreasing water-depth patterns for LCBD and LCBD_Repl_ (i.e., species replacement), while increasing patterns for total beta diversity and turnover, implying that total beta diversity and LCBD were dominated by species turnover or LCBD_Repl_. Further, bacteria showed a strong correlation with archaea regarding LCBD, total beta diversity and turnover. Such parallel patterns among bacteria and archaea were underpinned by similar ecological processes like environmental selection. Total beta diversity and turnover were largely affected by sediment total nitrogen, while LCBD and LCBD_Repl_ were mainly constrained by water NO_2_^−^-N and NO_3_^−^-N. For fungal community variation, no significant patterns were observed, which may be due to different drivers like water nitrogen or phosphorus. Taken together, our findings provide compelling evidences for disentangling the underlying mechanisms of community variation in multiple aquatic microbial taxonomic groups.

## Introduction

Lake level or water depth of lakes, especially in arid regions, is vulnerable to global change largely since the accelerated decreasing winter ice cover and increasing surface temperature have been caused by climate warming (Woolway et al., 2020). Lake ecosystems support a global heritage of biodiversity and helps sustaining a variety of ecosystem functions (Abell et al., 2008). Accordingly, understanding biodiversity-ecosystem functioning relationships (Wang et al., 2022) and disentangling the mechanisms of biodiversity variation, are crucial to predict aquatic ecosystem responses to global climate changes (Martiny et al., 2011). As an essential biodiversity facet, beta diversity is well applied to reveal the patterns from local to global scales (Socolar et al., 2016) in explaining the assembly mechanisms of community assemblages (Mena and Vázquez-Domínguez, 2005). More generally, biological community variation along environmental gradients and the underlying drivers have been examined across temporal (Hatosy et al., 2013) and spatial scales (Wang et al., 2013), such as in mountain streams (Wang et al., 2020) and plateau lakes (Yuan et al., 2021). However, it is still relatively rare for addressing water-depth patterns of beta diversity and its components (i.e., turnover and nestedness; Baselga, 2010) across taxonomic groups, especially regarding aquatic microbes in semi-arid lakes.

Since the term “beta diversity” was introduced to describe biological community variation by Whittaker in 1960 (Whittaker, 1960), numerous methods have been proposed to disentangle its underlying mechanisms. In recent studies, for beta diversity, its partitioning components of turnover and nestedness mostly lie in the heart of discussion (Soininen et al., 2018). There are two alternative approaches for unraveling the variation in beta diversity components, that is beta diversity partitioning developed by Baselga (2010) and the Local Contributions to Beta Diversity (LCBD) partitioning by Legendre and De Cáceres (2013). According to the Baselga’s framework, beta diversity (i.e., Sørensen dissimilarity) can be partitioned into two components: species turnover and nestedness (Baselga, 2010). The former indicates that one species replaces another without changing species richness, while the latter refers to the richness differences attributable to species gain or loss (Baselga, 2010). More precisely, species turnover can effectively reflect species sorting *via* ecological processes like environmental selection or dispersal limitation, whereas nestedness is largely relevant to the dynamic processes of the ordered extinction-colonization (Si et al., 2016). Given that the ecological processes underpinning community assembly can be revealed by species turnover and nestedness (Meynard et al., 2011), partitioning beta diversity may provide a better comprehension for the structuring of biological communities at spatial scales or along environmental gradients (Medeiros et al., 2016).

Unlike the Baselga’s method (Baselga, 2010), LCBD shows a shorter history of evaluating variation in community composition and can substantially characterize the degree of community uniqueness (Szabó et al., 2019; Wang et al., 2020). Based on the dissimilarity measures like Sørensen indices, total LCBD can be divided into the components of species replacement (i.e., LCBD_Repl_) and nestedness (i.e., LCBD_Nes_; Legendre, 2014). The two partitioned components allow us to reveal the underlying mechanisms that guide the structuring of sites’ uniqueness for biological communities, which can largely facilitate the local biota to make corresponding conservation strategies. Ecologically, LCBD is often applied to quantify the relative contributions of individual sampling sites to overall beta diversity (Legendre and De Cáceres, 2013). For example, in the subarctic ponds of Finland and Norway, the U-shaped elevational patterns in LCBD have been unraveled in bacterial communities, which provides novel perspectives for the complex biogeography patterns of microbial taxa (Yeh et al., 2019). Additionally, there exists different water-depth patterns in the total LCBD, LCBD_Repl_, and LCBD_Nes_ from bacteria, diatoms to chironomids in a deep lake, which is largely driven by different environmental factors, such as spatial or biotic variables (Wu et al., 2020). Notably, LCBD and Sørensen coefficients can substantially provide crucial insights into the ecological processes driving the community assemblages (Castro et al., 2019; Wu et al., 2020). Therefore, coupling the two above approaches by Baselga and Legendre may pave a better way to examine the structuring of aquatic microbial communities.

Here, based on the Baselga’s and Legendre’s frameworks (Baselga, 2010; Legendre and De Cáceres, 2013), we partitioned the two beta diversity metrics Sørensen dissimilarity (i.e., total beta diversity) and LCBD into species turnover (or LCBD_Repl_) and nestedness (or LCBD_Nes_), and then examined the water-depth patterns in beta diversity components regarding bacteria, archaea and fungi in surface sediments of the semi-arid lake Hulun Lake in the Northern China. We primarily focused on the following three aims: First, to reveal the underlying mechanisms of variation in total beta diversity and LCBD in bacterial, archaeal and fungal lake sediment communities. Second, to investigate the cross-taxon congruence among bacteria, archaea and fungi regarding the above two metrics and their partitioning components. Third, to test the relative importance of water and sediment variables on total beta diversity and LCBD across the three microbial groups and evaluated how each driving factor influences the loss and replacement of species along the water depth.

## Materials and methods

### Study area and field sampling

Hulun Lake (48°33′–49°20′ N, 116°58′–117°48′ E), also known as Dalai Lake, located in the west of Hulunbuir Prairie, is a shallow semi-arid lake in the Mongolian Plateau (Qu et al., 2019). As the fifth largest freshwater lake in China, it has a surface area of 2,315 km^2^, a perimeter of 447 km, a maximum depth of ~8.0 m, a mean depth of ~5.7 m, and the water storage capacity of 13.2 billion m^3^ (Li et al., 2019). Hulun Lake, a historic and tectonic lake, showing an irregular or unique rectangular shape with the length of 93 km and the largest width of 41 km (Li et al., 2019), lies in the semi-arid region of the middle temperate zone (Wen et al., 2010). Accordingly, the lake is strongly affected by the temperate continental climate, displaying mean annual air temperature of −0.2°C, precipitation of 290 mm, and evaporation of 1,600 mm (Xie et al., 2015). Additionally, its water supplies are mainly fed by direct surface runoff, rainfall and groundwater, and Kherlen River and Urson River provide the main source of water for this lake (Li et al., 2019). In the past 20 years, Hulun Lake has experienced dramatic water level fluctuations mainly resulting from variations in river runoff and evaporation associated with climate change (Fu et al., 2021). To date, Hulun Lake is under meso-eutrophic with a documented history of severe eutrophication (Zhai et al., 2011), such as cyanobacteria blooms (Liang et al., 2016).

In June 2020, surface-sediment samples (0–5 cm) were collected from 19 sites of Hulun Lake (Supplementary Figure S1) using a stainless-steel grab sampler. At each sampling site, these surface sediments were fully stirred and homogenized, and then transferred to sterilized bottles. Meanwhile, 100 ml surface water was sampled within 50 cm. These sediment and water samples were transferred to laboratory at-20°C. Sediment samples were dried using a vacuum freeze dryer and then stored in-20°C before the physicochemical and biological analyses. Water depth was measured and recorded *in situ*.

### Measuring the water or sediment properties

For environmental factors of surface water, temperature, dissolved oxygen (DO), pH and electrical conductivity (EC) were measured *in situ* using a YSI 650 multi-parameter display system with a 600XL probe (Wu et al., 2020). Secchi disk depth (SD) is recorded *in situ* to indicate the water transparency. Additionally, NO_3_^−^-N, NO_2_^−^-N, NH_4_^+^-N, total nitrogen (TN), total phosphorus (TP) and PO_4_^3−^-P were determined based on the standard methods (Huang et al., 2000).

For sediment properties, we determined pH, EC, water content (WC), grain size (GS), dissolved organic carbon (DOC), NO_2_^−^-N, NO_3_^−^-N, NH_4_^+^-N, PO_4_^3−^-P, TN, TP, and total carbon (TC). Note that pH and EC were measured *in situ*. WC was measured by oven dry method and pycnometer method (Wang et al., 2007). Sediment samples were first dissolved using ultrapure water and then filtrated with the 0.45 μm membrane to obtain the aqueous solution. NO_2_^−^-N, NO_3_^−^-N, NH_4_^+^-N, and PO_4_^3−^-P were then measured *via* the same methods as water samples (Huang et al., 2000). DOC was extracted according to our previous studies (Zhang et al., 2021), and measured by a total organic carbon analyzer (ET1020A, United States). The freeze-dried sediment samples were ground into fine powder and passed through a 100-mesh sieve for TC and TN analyses using an elemental analyzer (Flash EZ 1112 series, Italy), and for TP measurement using molybdenum blue colorimetry after acidification (Sparks et al., 1996). GS32 (<32 μm) was selected as a representative of GS, and the detailed measurement or calculation methods for GS are described in a previous study (Wang et al., 2012).

### Microbial communities

Briefly, as previously described (Wang et al., 2013; Yuan et al., 2021), the genomic DNA was extracted from 0.45 g of frozen sediments using the DNeasy^®^ Power Soil Kit (QIAGEN, Germany). Its quality was tested *via* NanoDrop One/OneC UV–Vis spectrophotometer (Thermo Fisher Scientific, United States). For bacteria and archaea, the *16S rRNA* genes were amplified using the universal primers: 515F (5′-GTGYCAGCMGCCGCGGTAA-3′) and 806R (5′-GGACTACNVGGGTWTCTAAT-3′; Apprill et al., 2015; Parada et al., 2016). For fungi, we selected the gITS7F/ITS4R primer pair (gITS7F, GTGARTCATCGARTCTTTG, ITS4R, TCCTCCGCTTATTGATATGC; Ihrmark et al., 2012) to amplify the internal transcribed spacer 2 (*ITS2*) region of the rRNA gene. The *16S rRNA* and *ITS* amplicons were pooled independently and sequenced using Illumina HiSeq 2500 platform (Illumina, San Diego, United States). For the raw data, sequences analysis was then performed through Quantitative Insights into Microbial Ecology (QIIME) pipeline (v1.9.0; Caporaso et al., 2010b). Based on the seed-based UCLUST algorithm, the sequences with ≥97% similarity were regarded as the same operational taxonomic units (OTUs; Edgar, 2010). During the clustering, Singletons and Chimera sequence were excluded or removed by ChimeraSlayer (Haas et al., 2011). For each representative sequence, the Greengenes database was applied to align taxonomic information *via* PyNAST (Caporaso et al., 2010a). More details are described in previous studies (Zhou et al., 2016). After taxonomic assignment, for the following analyses, the communities of bacteria, archaea and fungi were rarefied at 65,800, 200, 66,300 sequences, respectively. The raw data sequencing 16S rRNA and ITS genes have been submitted to the NCBI Sequence Read Archive database and are available under accession numbers SRR13611586 to SRR13611623.

### Statistical analyses

For each sediment microbial group, we first explored the relationships between the relative abundances of their dominated phyla and water depth using linear and quadratic models. The water-depth patterns of the first three dominant phyla were visualized, which were *Proteobacteria*, *Chloroflexi* and *Acidobacteria* for bacteria, *Crenarchaeota*, *Euryarchaeota*, and *Parvarchaeota* for archaea, and *Ascomycota*, *Rozellomycota*, and *Chytridiomycota* for fungi. The better model was performed based on the lower value of Akaike’s information criterion (AIC; Yamaoka et al., 1978).

Second, according to the Legendre’s method (Legendre, 2014), the Local Contributions to Beta Diversity (LCBD) and its components (i.e., LCBD_Repl_ and LCBD_Nes_) were applied to estimate the degree of ecological uniqueness regarding the community composition. The LCBD was examined using the Sørensen-based indices of the Baselga’s family, and its indices were calculated *via* the function “LCBD.comp” based on the species replacement or nestedness matrices (Wu et al., 2020). The water-depth patterns of these LCBD indices were then explored using linear and quadratic models. Given the lowest value of AIC (Yamaoka et al., 1978), we obtained the best model.

Third, as proposed by Baselga (2010), we explored the depth-related patterns of the total beta diversity and its partitioned components such as species turnover and nestedness for the three taxonomic groups. Specifically, this method needs to be based on the multi-site different dissimilarity coefficients as follows: (1) the total beta diversity was employed *via* the Sørensen coefficient, (2) the species turnover was computed using Simpson coefficient, and (3) the nestedness was calculated with nestedness coefficient (Baselga, 2010; Gutiérrez-Cánovas et al., 2013). Moreover, the water depth difference was computed with the Euclidean distance. Analogous to LCBD, we then explored the relationship between the total beta diversity or its components and the water depth difference with linear and quadratic models. Mantel test (9,999 permutations; Mantel, 1967) was then performed to estimate the significance.

Further, we also explored the associations among bacteria, archaea and fungi using linear and quadratic models. For the three taxonomic groups, Pearson correlation was then applied to examine the associations between the environmental factors (i.e., water and sediment physiochemical properties) and the total LCBD or its components. Moreover, we performed Mantel test (Mantel, 1967) to explore the relationships between the abiotic variables and the total beta diversity or its partitioned components. Further, the multicollinearity (Supplementary Figure S4) between all explanatory variables was evaluated using the function “varclus” based on the R package Hmisc, and then one factor was selected from the variables having high correlation coefficients (Spearman r > 0.7; Wang X.-B. et al., 2017).

Then, the random forest model (Feld et al., 2016) was employed to examine the most important predictors of the LCBD and its two components from the water and sediment variables above. Notably, we applied cross-validation (Elith et al., 2008) to get the optimal number of 2,000 trees. The importance of explanatory variables was valuated based on its frequency of selection (for splitting), weighted by a measure of improvement in the model given each split and averaged across all the trees (the contributions were scaled to sum to 100; Wu et al., 2020).

Finally, we applied the multiple regression on distance matrices (MRM; Lichstein, 2007) to estimate the relationships between the above environmental variables and each component of beta diversity for the three taxonomic groups. These variables were z-score standardized (i.e., mean = 0, SD = 1) before performing the statistical analyses. There were two groups of explanatory variables: water and sediment variables, which were calculated as a Euclidean distance matrix. Additionally, the spurious associations among the variables were excluded using the above function “varclus” (Wang X.-B. et al., 2017). Then, the non-significant variables were removed from the initial MRM test and the test was further re-ran. The matrix permutation was performed 999 times to examine the significance of the partial regression (Wu et al., 2020). These above analyses were implemented with the R packages vegan V2.5–4 (Borcard and Legendre, 2002), betapart V1.5.1 (Baselga et al., 2018), ecodist V2.0.1 (Goslee and Urban, 2007), Hmisc (Harrell, 2019), and randomForestSRC V2.9.0 (Liaw and Wiener, 2002).

## Results

### Relationship between microbial dominant phyla and water depth

**Figure 1:**
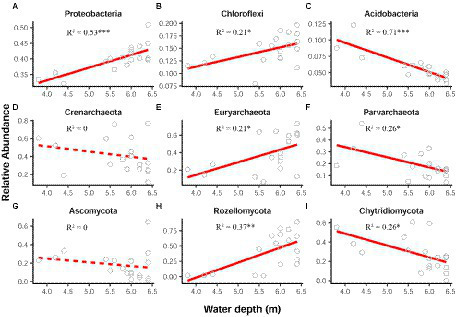
Relative abundance of the dominant bacterial **(A–C)**, archaeal **(D–F)**, and fungal **(G–I)** phyla along the water-depth gradient. The relationships between the water depth and relative abundance were modeled using linear and quadratic models, and the best model was selected based on the lowest value of Akaike’s information criterion. Solid and dashed lines denote the significant and non-significant relationships, respectively. ^*^p ≤ 0.05; ^**^p < 0.01; ^***^p < 0.001.

In general, the relative abundance of bacterial, archaeal and fungal communities at phylum level showed different water-depth patterns (Figure 1). In this lake, bacterial community was dominated by the phyla *Proteobacteria*, *Chloroflexi*, and *Acidobacteria,* comprising about 40%, 15%, and 6%, respectively (Supplementary Figure S2A). Note that the relative abundance of *Proteobacteria* and *Chloroflexi* significantly (*p* < 0.05) increased toward deep water (Figures 1A,B), while *Acidobacteria* declined (*p* < 0.001; Figure 1C).

Regarding archaeal community, the dominant phyla were *Crenarchaeota* (40%), *Euryarchaeota* (40%), and *Parvarchaeota* (20%), which were mostly predominant in the whole lake (Supplementary Figure S2B). Notably, for the relative abundance, *Euryarchaeota* exhibited a significant (*p* < 0.05) increasing pattern along the water depth (Figure 1E), whereas *Parvarchaeota* showed a decreasing pattern (Figure 1F).

Additionally, fungal community was mainly composed of the phyla *Rozellomycota*, *Chytridiomycota* and *Ascomycota,* comprising about 41%, 28%, and 18%, respectively (Supplementary Figure S2C). The relative abundance of *Rozellomycota* significantly (*p* < 0.01) increased with water depth (Figure 1H), while that of *Chytridiomycota* decreased (*p* < 0.05) toward deep water (Figure 1I).

### Patterns and drivers of LCBD and its components along the water depth

**Figure 2:**
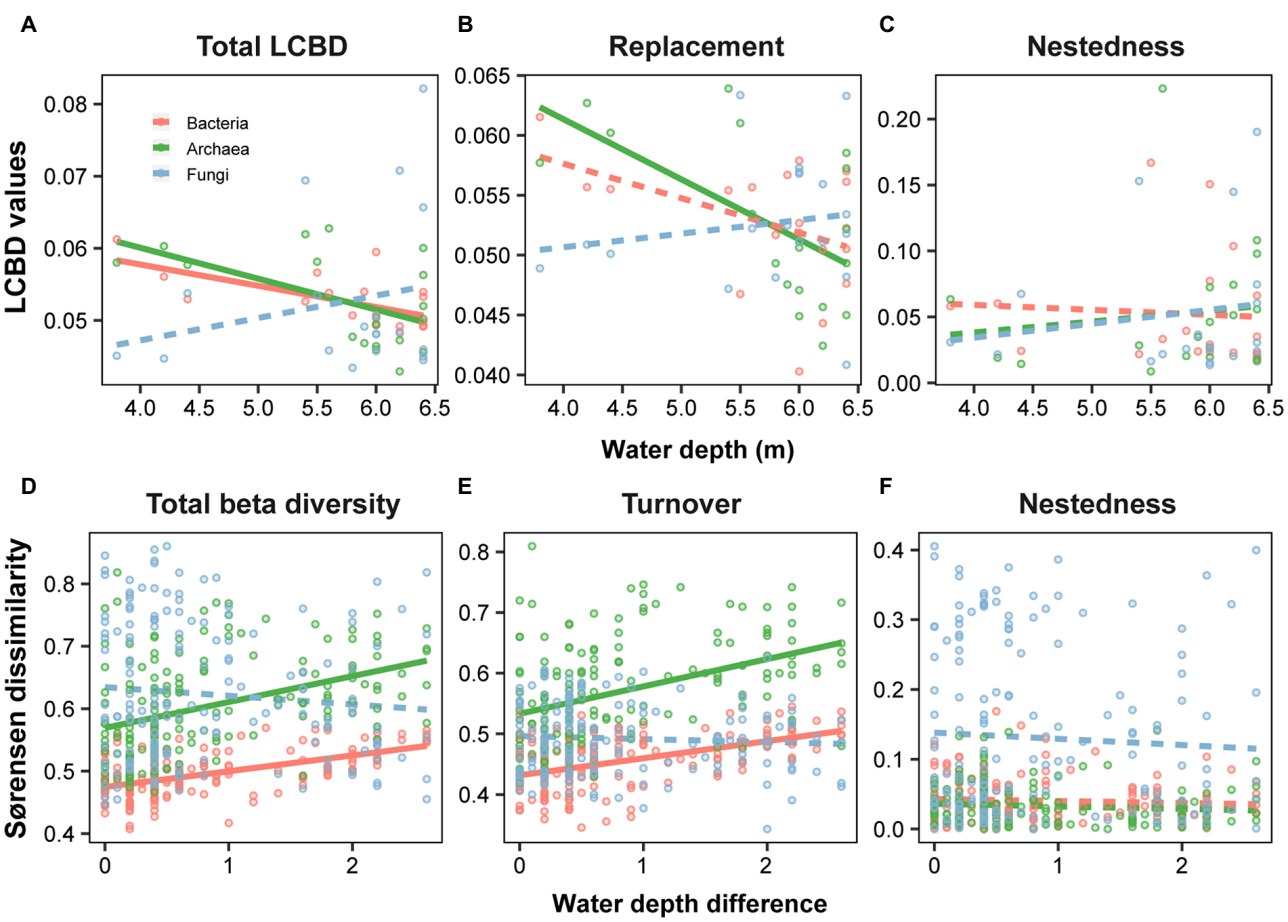
Water-depth patterns of LCBD and total beta diversity and their components for bacteria, archaea and fungi. We considered two metrics for the variance in community composition, that is the local contributions to beta diversity (LCBD) and the total beta diversity. The LCBD **(A)** was divided into species replacement **(B)** and nestedness **(C)**, and the total beta diversity **(D)** was partitioned into turnover **(E)** and nestedness **(F)**. These metrics and components were linearly or quadratically modeled against the water depth or water depth distance, and solid and dashed lines indicate the significant and non-significant relationships, respectively. More details on these models are listed in Supplementary Table S2.

The relationship between LCBDs (i.e., total LCBD, LCBD_Repl_, and LCBD_Nes_) and water depth was distinct among the three microbial groups. For total LCBD, the water depth pattern with a significant (*p* < 0.05) decrease was observed between bacteria and archaea (Figure 2A). The latter had the stronger variation in total LCBD toward deep water, with a slope of −0.0043, whereas the former showed the lower slope at −0.0030 (Figure 2A; Supplementary Table S2). For LCBD_Repl_, there was a significant (*p* < 0.05) decreasing pattern for archaea, while not significant for bacteria (Figure 2B). Meanwhile, for LCBD_Nes_, there was non-significant depth-related pattern for bacteria and archaea along the water-depth gradient (Figure 2C). Note that such non-significant patterns were also observed for all of fungal LCBDs, including the total LCBD, LCBD_Repl_, and LCBD_Nes_ (Figures 2A–C).

**Figure 3:**
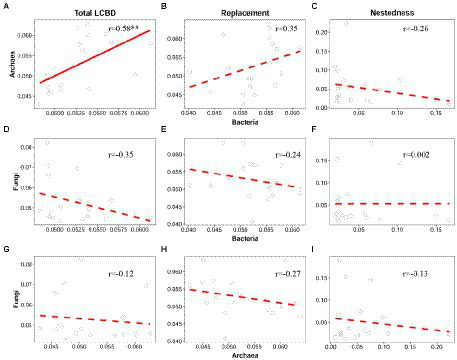
Correlation between bacteria, archaea, and fungi regarding the total LCBD and its two components. For the total LCBD **(A,D,G)**, species replacement **(B,E,H)**, and nestedness **(C,F,I)**, the relationships between bacteria, archaea, and fungi were modeled with linear and quadratic models, and the best model was selected based on the lowest value of Akaike’s information criterion. The solid and dashed lines indicate the significant and non-significant relationships, respectively. ^*^p ≤ 0.05; ^**^p < 0.01; ^***^p < 0.001.

As expected, bacteria had a high positive correlation with archaea regarding the total LCBD (Pearson *r* = 0.58, *p* < 0.01) and LCBD_Repl_ (*r* = 0.35, *p* > 0.05), although their correlation was not significant for LCBD_Repl_ (Figures 3A,B). Correspondingly, bacteria and archaea both showed consistent and non-significant correlations with fungi regarding the total LCBD and LCBD_Repl_ (Figures 3D,E,G,H). Likewise, for LCBD_Nes_, there was still no significant correlation among all of the three taxonomic groups (Figures 3C,F,I).

**Figure 4:**
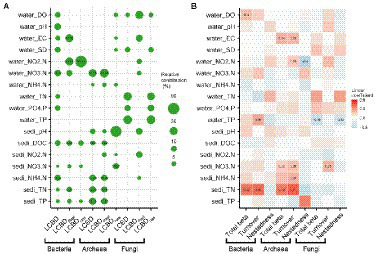
The water and sediment factors related to the variance in community composition. The local contributions to beta diversity (LCBD) are divided into species replacement (i.e., LCBD_Repl_) and nestedness (i.e., LCBD_Nes_), and the total beta diversity (Total beta) is partitioned into turnover and nestedness. Random forest analysis **(A)** was applied to show the relative contributions of the water and sediment factors in explaining the LCBD and its two components. The multiple regression on distance matrices **(B)** was performed to explain the total beta diversity and its partitioned two components by these abiotic variables. The size of the circle and the numerical value indicate the relative contribution, and the color or the numerical value denote the linear coefficients. Moreover, the presence of these numerical values also indicates the significant (*p* < 0.05) relationship. The abbreviations of these variables are listed in Supplementary Table S1.

Further, the importance of each environmental variable on the total LCBD, LCBD_Repl_ and LCBD_Nes_ varied substantially with the different microbial taxa. For instance, for bacteria, water NO_3_^−^-N and sediment NH_4_^+^-N exerted great influences on the total LCBD, with the relative contribution of 16.12% and 14.87%, respectively, while water NO_2_^−^-N was the most important variable explaining the variation in the LCBD_Repl_ and LCBD_Nes_ with the relative contribution of 33.15% and 90.61%, respectively (Figure 4A). For archaea, the total LCBD and LCBD_Repl_ were both well explained by water NO_3_^−^-N and sediment TP, TN, and NH_4_^+^-N, whereas the LCBD_Nes_ was primarily affected by sediment NO_3_^−^-N with the relative contributions of 14.87% (Figure 4A). Conversely, for fungal LCBDs, partial environmental factors showed certain influence (Figure 4A) but no significant correlation (Supplementary Figure S3A).

### Water-depth patterns and drivers of the total beta diversity and its components

Patterns in total beta diversity and its components along water depth difference were interesting across microbial taxonomic groups. For the total beta diversity, there were linearly positive and significant (*p* < 0.05) relationships with water depth changes for bacteria and archaea (Figure 2D), and the latter changed faster than the former with the decay slopes of 0.0414 and 0.0253, respectively (Figure 2D; Supplementary Table S2). Similarly, for the turnover component, bacteria and archaea both showed significant (*p* < 0.05) increasing patterns with larger water-depth difference (Figure 2E). Note that such water-depth decay patterns were not significant for nestedness components of bacterial and archaeal total beta diversity (Figure 2F). In addition, for fungal community, there was still non-significant depth-related pattern for total beta diversity and the two partitioning components, which is similar to the LCBDs (Figures 2D–F).

**Figure 5:**
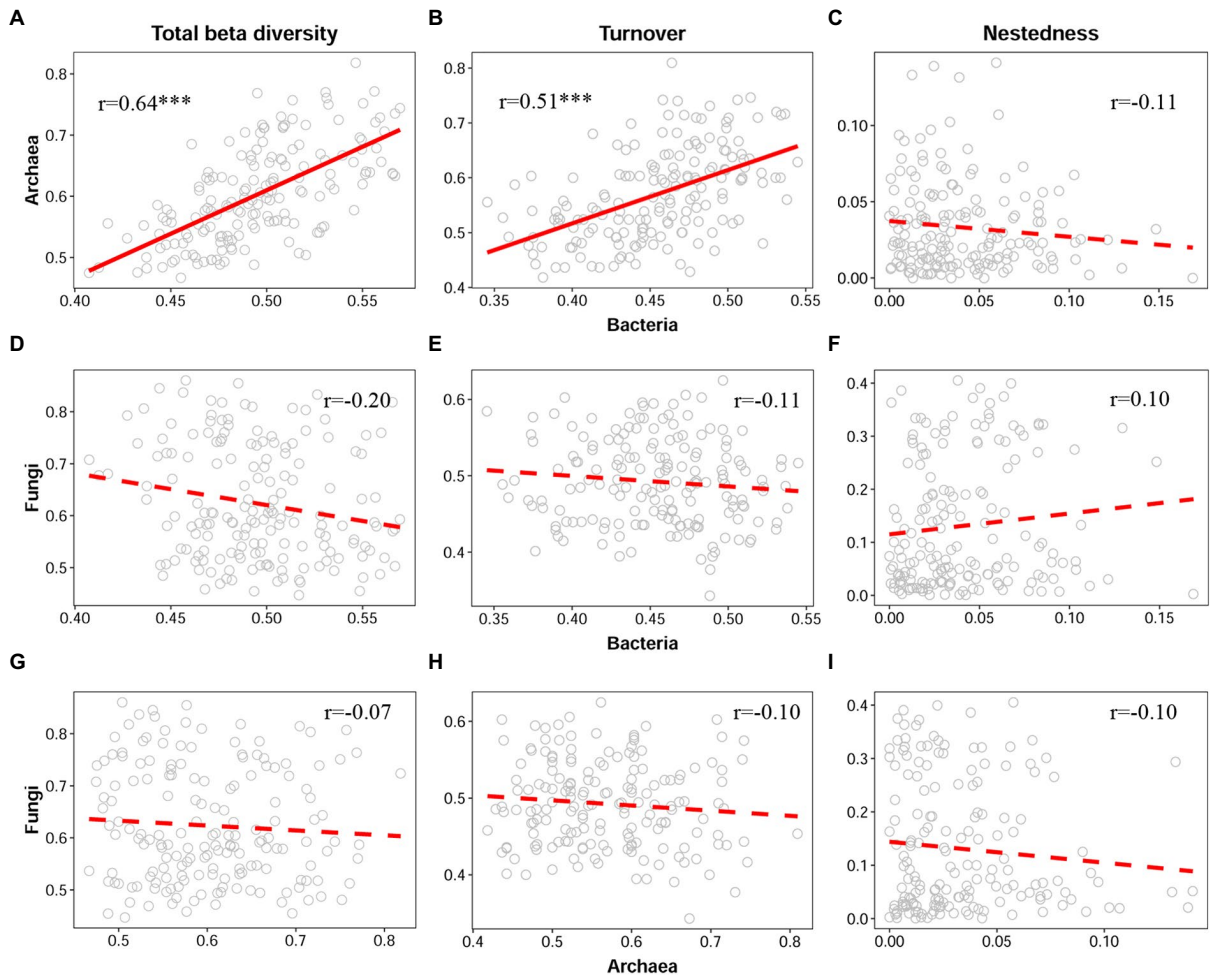
Correlation between bacteria, archaea and fungi regarding the total beta diversity and its two components. For the total beta diversity **(A,D,G)**, turnover **(B,E,H)**, and nestedness **(C,F,I)**, the relationships between bacteria, archaea, and fungi were modeled using linear and quadratic models, and the best model was selected based on the lowest value of Akaike’s information criterion. The solid and dashed lines indicate the significant and non-significant relationships, respectively. ^*^p ≤ 0.05; ^**^p < 0.01; ^***^p < 0.001.

When viewed among the three microbial groups, intriguingly, there were cross-taxon congruence for the total beta diversity and its components. Accordingly, bacteria showed a strong positive correlation with archaea regarding total beta diversity (Mantel *r* = 0.64, *p* < 0.001, Figure 5A) and the turnover component (Mantel *r* = 0.51, *p* < 0.001, Figure 5B). Moreover, similar to the LCBD, fungi were still not significantly correlated with bacteria and archaea regarding total beta diversity (Figures 5D,G) and the species turnover (Figures 5E,H). For the nestedness component, there was still no significant correlation from bacteria, archaea to fungi, which is in line with the LCBD_Nes_ (Figures 5C,F,I).

It should be noted that the importance of water and sediment variables to total beta diversity and its partitioning components also varied across the three groups. For bacteria and archaea, sediment TN was the most important (*p* < 0.05) predictor of the total beta diversity and its turnover component (Figure 4B). Additionally, the nestedness component of archaea was significantly (*p* < 0.05) negatively correlated with water NO_2_^−^-N, with the linear coefficient of −0.5 (Figure 4B). For fungi, total beta diversity and the nestedness component were mainly affected by water TP, while the turnover component was simply constrained by sediment NO_3_^−^-N (Figure 4B). However, there was no significant correlation between fungal total beta diversity and its components and each variable except for water PO_4_^3−^-P (Supplementary Figure S3B).

## Discussion

Partitioning beta diversity is an effective way to unravel the response mechanism of organisms to climate change, especially in climate-sensitive arid regions (Wang J. et al., 2017). Turnover and nestedness, showing different implications for biodiversity conservation (Medeiros et al., 2016), are often invoked to disentangle the spatial patterns of compositional beta diversity in biogeography and microbial ecology (Soininen et al., 2018). In the semi-arid Hulun Lake, we investigated the driving mechanisms of beta diversity and community uniqueness for bacteria, archaea and fungi toward deeper water based on the Baselga’s (Baselga, 2010) and Legendre’s (Legendre, 2014) frameworks, respectively. Our results suggest that (1) the relative abundance of most dominant phyla had significant water-depth patterns across the three taxonomic groups. (2) For the Local Contributions to Beta Diversity and the total beta diversity and its turnover component, water-depth patterns were significantly observed for bacteria and archaea, but not for fungi. (3) There was a high correlation between bacteria and archaea regarding the LCBD, the total beta diversity and its turnover components. (4) The relative importance of water and sediment environmental factors on the LCBD, total beta diversity and their additive components was demonstrated to vary for the studied microbial taxonomic groups.

### Water-depth patterns of the LCBD, total beta diversity and their partitioned components

For the total LCBD, we found that bacteria and archaea both showed significant decreasing patterns along the water depth, while fungi did not (Figure 2A). Such variation may be relevant to sites with special ecological conditions like species combinations and environmental changes. Previous studies have shown that sites with high LCBD values not only have unique species combinations (da Silva et al., 2018), but also indicate human interference or higher proportions of allochthonous species (Landeiro et al., 2018). As the water level fluctuates, indeed, shallower sites are most likely the consequence of land flooding or mean annual precipitation and water evaporation, which largely changes the habitat environment of autochthonous species and thereby presents high local contribution to beta diversity. Intriguingly, such variations are also observed for bacterial and archaeal communities in semi-arid grassland soils, which reveals that higher precipitation can effectively regulate microbial assemblies and strengthen their interactions in water-limited areas (Wang et al., 2018). For LCBD_Repl_, such decreasing water-depth patterns are also observed among bacteria and archaea, albeit not significant for bacteria (Figure 2B). To our knowledge, this is the first study that such parallel patterns in LCBD and its components are observed across microbial taxonomical groups, implying that bacterial and archaeal community uniqueness may be affected by similar ecological progress (Shade et al., 2018; Rapacciuolo et al., 2019) such as environmental selection. Notably, while bacteria and archaea responded to the variation in water depth, fungi unexpectedly showed non-significant patterns toward deep water regarding LCBD, LCBD_Repl_, and LCBD_Nes_. During the historical events induced by drought, eukaryotic communities including protist and fungi generally present strong resilience in freshwater ecosystems (Simon et al., 2016); that is, fungal assemblages in the dry sediment can effectively restore community structure after water refill. Compared to bacteria and archaea, there is thus lower proportions of allochthonous species (i.e., species gain or loss) for fungal communities in this semi-arid lake.

Previous studies have reported that species turnover is crucial for disentangling the underlying mechanism of beta diversity in aquatic ecosystem (Epele et al., 2019). Likewise, for bacteria and archaea, our results indicated that the total beta diversity and its turnover component had consistent increasing patterns along water-depth difference (Figures 2D,E), which largely consolidates the fact that species turnover contributes to the total beta diversity for aquatic microbial taxa. Moreover, such predominance is also observed in species of other lake environments. For instance, for bacterioplankton, turnover shows a higher contribution to total beta diversity than nestedness in the 25 shallow lakes of southern Brazil (Lima et al., 2020). It should be noted that when considering some specific spatial factors such as geographical distance between lakes, the predominance of bacterioplankton turnover may be replaced by nestedness (Lima et al., 2020). In the Grand Galibier Massif of the French South-Western Alps, however, high turnover is exhibited in Crenarcheal, bacterial and fungal community distribution, which is greatly associated with plant species composition but not geographical distance (Zinger et al., 2011). Additionally, regarding nestedness, our studies showed no significant patterns for bacteria, archaea and fungi (Figure 2F). In particular, for fungi, there was no significant depth-related pattern in total beta diversity and its two components. This may be because fungi have large difference with bacteria and archaea regarding biological characteristics, such as resilience and trophic position. Most importantly, nestedness is well applied to indicate dispersal limitation, while turnover can be as an indicator of species sorting based on environmental filters of microbial communities (Lima et al., 2020). Accordingly, for the total beta diversity and turnover component, such parallel pattern between bacteria and archaea may be governed by similar ecological progress (Shade et al., 2018; Rapacciuolo et al., 2019) like environmental selection.

In addition, we found that there is a strong correlation between bacteria and archaea regarding the total beta diversity and its turnover component (Figures 5A,B), which further underpins the above synchrony among bacteria and archaea toward deep water. As previously reported, such cross-taxon congruence can occur if different organisms covary in space regarding alpha or beta diversity (Rooney and Azeria, 2015). Notably, such synchrony is also observed in macroorganisms. For example, in the Espinhaç Range and Mantiqueira Range of southeastern Brazil, total beta diversity of both galling insects and host plant show similar patterns along the elevational gradients, mainly driven by turnover rather than nestedness (Coelho et al., 2018). Intriguingly, for bacteria and archaea, such congruence is also found for the total LCBD (Figure 3A). This may reflect the fact that beta diversity (including total beta diversity and LCBD) in semi-arid lake ecosystems is taxonomically dependent among bacteria and archaea. Our findings supported the conclusion that the total beta diversity of two high-dependent taxa is contributed by a similar component (i.e., turnover). It should be noted that, consistent with Yeh et al. (2019), no correlation was significantly detected between fungi and other microbial taxa, regardless of total beta diversity or LCBD. Conversely, in an arid inland river basin of northwest China, soil bacteria shows a high correlation with fungi for the total beta diversity and species turnover, and meanwhile, both of them respond sensitively to variations in such environmental variables and geographic distances (Wang J. et al., 2017). Accordingly, the relationship between sediment fungi and bacteria or archaea in this lake may be related to environmental variation.

### Environmental determinants of the LCBD, total beta diversity and their components

For bacteria and archaea, we found that variations in total LCBD and LCBD_Repl_ were greatly contributed by the water factors such as NO_3_^−^-N or NO_2_^−^-N (Figure 4A), which may be related to eutrophication and the local climates. Hulun Lake, as a large hypereutrophic steppe lake, has clearly shown that microbial taxa such as bacteria and archaea frequently participate in the cycling of carbon, nitrogen and phosphorus in aquatic ecosystem (Shang et al., 2020). Over the past few years, however, the warming and drying climate has gradually accelerated the shrinking of water area and the decrease of water level in Hulun Lake (Cai et al., 2016). These climate changes have constantly caused the variations in water or sediment factors, and further driven the site uniqueness of microbial communities. The resulting water depth variation has a nonnegligible effect on determining environmental factors in lacustrine ecosystems (Wu et al., 2020). For example, in Lake Azul of São Miguel Island, environmental variables such as light intensity, nutrient availability or disturbance regimes vary largely with water depth, and thereby resulted in the distinct distribution of biological assemblages (Raposeiro et al., 2018). Considering the vertical variations of light, nutrients and other physical context, water depth is well accepted to be a better determinant driving the site uniqueness of bacterial and diatom communities in Lugu Lake (Wu et al., 2020). Consistent with this, for bacteria and archaea, our results revealed a strong negative correlation between water depth and the total LCBD or LCBD_Repl_ (Supplementary Figure S3A), implying that the drivers of bacterial and archaeal community uniqueness could be substantially constrained by water depth. In addition, climate change has a profound impact on the terrestrial input of inorganic nutrients and organic matter in aquatic ecosystems, thereby increasing the rate of eutrophication for water bodies (Moss et al., 2011). In particular, drought, as an extreme hydrologic event, will accelerate the occurrence of eutrophication when nutrients are overenriched (Paerl et al., 2018; Walter et al., 2018). As noted above, the uniqueness of bacterial and archaeal communities may be predominantly caused by eutrophication and climatic change.

Contrary to the LCBD, our findings revealed that bacterial and archaeal total beta diversity and turnover component were mainly influenced by sediment factors like TN rather than water factors (Figure 4B). As such, the observed total beta diversity and species turnover may be associated with the trophic status or nutrient supply. Although numerous nutrients and organic matters are stored in sediments of lakes and wetlands (Tranvik et al., 2009), much less is known about the effect of nutrients on the total beta diversity and its components across microbial taxa. In the shallow lakes of the southern Brazil, high species turnover is observed in bacterioplankton, strongly conditioned by environmental factors such as TN and total dissolved nitrogen (Lima et al., 2020). Additionally, nutrients, including water NH_4_^+^-N, NO_3_^−^-N and NO_2_^−^-N, show great direct effects on bacterial species turnover in aquatic microcosms, and such turnover rates do not decrease with lower temperatures (Wang et al., 2016). Admittedly, nutrient status is closely related to the sustainability of lake ecosystems, and meanwhile, the growths of aquatic organisms are largely governed by nitrogen concentrations such as TN, NO_3_^−^-N, NO_2_^−^-N and NH_4_^+^-N (Chuai et al., 2012). Collectively, the total beta diversity and species turnover of bacteria and archaea were well explained by sediment TN, even after accounting for the water variables.

Regarding the LCBD, total beta diversity and their components, fungi have almost nonsignificant water-depth patterns and drivers (Figures 2, 4), which may be related to its resilience in response to climate disturbances (e.g., precipitation or drought). As noted above, fungal assemblages in the dry sediment can effectively restore community structure after water refill (Simon et al., 2016). Indeed, fungi is more drought-tolerant than bacteria since its hyphae can effectively obtain water from the water-filled micropores (Boer et al., 2005). Based on such nonsignificant strong contribution from water factors like TP and TN (Figure 4), we speculated that fungal communities might be related to the trophic status or nutrient supply of lake water and was less disturbed by precipitation or drought.

### Perspectives

To better clarify these studies, there are three main perspectives for the interpretation. First, historical legacies may be the key factor underlying the water-depth patterns in beta diversity. As proposed by Cardoso (Cardoso et al., 2014), the observed patterns of biodiversity cannot be fully explained without considering the effects of historical factors, which can be reflected in the case of the invertebrate taxa (González-Trujillo et al., 2020a) and diatoms (González-Trujillo et al., 2020b) in the Orinoco river basin. In the Neotropical region, the distinct river forms and riparian ecosystems are shaped by past geological and climatic events, namely Andean uplifts and glacier retreats, which greatly affects the evolution of ecosystem and biological succession (Hoorn et al., 2010). Hulun Lake, having experienced the processes of swamping, drying, and sharp increase or decrease of water level, is constantly evolving between saline, brackish and freshwater lakes owing to the high evaporation (Li et al., 2006). With special geographical location of high latitude, Hulun Lake shows a half-year frozen period and its salinity is 300–400 mg L^−1^ higher than that of unfrozen status (Li et al., 2006). Under the interplay of natural factors and anthropogenic activities (Li et al., 2019), Hulun Lake shows a long history documenting the frequent outbreaks of severe eutrophication (Zhai et al., 2011), such as the cyanobacteria bloom (Liang et al., 2016), which largely influences the nutrient status of this lake. Due to the historical legacies, nutrients (Bowen et al., 2009) and salinity (Awata et al., 2012) have crucial roles in shaping microbial structure in aquatic ecosystems. Therefore, for microbial taxa, such water-depth pattern in beta diversity may be affected by historical factors.

Second, climate is a pivotal driver of biodiversity patterns (Gaston, 2000), which is widely observed in bacterial biogeographic patterns in arid and semi-arid grasslands (Wang et al., 2015; Wang J. et al., 2017). Hulun Lake, as a semi-arid lake, affected by the temperate continental climate (Xie et al., 2015), is exceptionally fragile and suffers frequently from extreme climatic phenomena such as dramatic drought and low temperatures. Notably, drought intensification and precipitation alterations can effectively drive the turnover of composition structures, which is well known in the case of soil microbiome (Fry et al., 2016) and stream invertebrates (Aspin et al., 2018). Moreover, species turnover is also considered as the legacy of climatic or geological events (Hazzi et al., 2018). For example, for the microbenthic communities in the Paraíba do Norte or Mamanguape estuaries, beta diversity is individually affected by species turnover in dry season, while turnover and nestedness in wet season (Medeiros et al., 2016). Furthermore, in response to temperature changes, organisms evolving in temperate zones generally have stronger thermal adaptations and dispersal capabilities than that in tropical regions (Janzen, 1967). Accordingly, compared to tropical zones, species turnover is often lower in temperate regions (Encalada et al., 2019). Even so, our results reveal that bacterial and archaeal total beta diversity were mainly explained by species turnover (Figure 2), implying that microbial communities may be relatively sensitive to climate change. Given that the role of climatic variation and dispersal ability on species turnover plays key importance (Dobrovolski et al., 2012), patterns in microbial beta diversity may be substantially relevant to climate changes.

Third, similar patterns are underpinned by equivalent ecological processes (Shade et al., 2018). Such processes, including drift, selection and dispersal, governs the turnover of biological community composition in space (Stegen et al., 2013). Simultaneously, turnover can replace species *via* environmental selection and historical or spatial restriction (Baselga and Orme, 2012), and its predominance can evaluate beta diversity across different taxa, type of dispersal or trophic position (Soininen et al., 2018). In our studies, the total beta diversity (Figure 2D) and turnover (Figure 2E) of bacteria and archaea consistently increased with the water depth difference, implying that their parallel patterns are supported by similar processes like environmental selection. Note that the same patterns are not only observed in total beta diversity and turnover, but also in LCBD and LCBD_Repl_. Thus, for bacteria and archaea, consistent patterns in LCBD and LCBD_Repl_ are also theoretically governed by same ecological processes such as environmental filtering. From macro-organisms to microbes, whether it is regional or local variation in community structures, such parallel patterns are dictated by similar processes (Rapacciuolo et al., 2019). Additionally, more research should be implemented to further disentangle the mechanisms underlying the parallel patterns across taxonomic groups, such as exploring other differential phylotypes in abundance and co-occurrence data. Thereby, parallel patterns can serve as a powerful tool for understanding the forces driving species turnover or replacement across taxonomic groups, even after accounting for the absence of theoretical derivations.

## Conclusion

Overall, based on the Baselga’s and Legendre’s approaches, our studies provide evidence that beta diversity including total beta diversity and LCBD was taxonomically dependent across microbial taxa in a semi-arid lake. There were significant water-depth patterns in total beta diversity, LCBD and their components for bacteria and archaea but not for fungi. For both of bacteria and archaea, the total beta diversity attributed to species turnover, and LCBD was predominated by LCBD_Repl_. Moreover, there was a spatial synchrony between bacteria and archaea regarding total beta diversity, species turnover, LCBD and LCBD_Repl_. Such parallel patterns are largely underpinned by similar ecological process like environmental selection. For instance, the total beta diversity and species turnover were better explained by sediment factors such as total nitrogen, while LCBD and LCBD_Repl_ were strongly affected by water factors like NO_2_^−^-N or NO_3_^−^-N. In addition, for fungi, there was no significant pattern in each beta diversity index toward deep water, which may be due to different drivers like water nitrogen or phosphorus. To date, studies partitioning beta diversity across multiple taxonomic groups are still relatively lacking in aquatic ecosystems. We encourage future studies to confirm our findings by comparing the associations among the multiple taxonomic groups in other aquatic ecosystems or under other climatic conditions.

## Data availability statement

The raw data sequencing 16S rRNA and ITS genes have been submitted to the NCBI Sequence Read Archive database and are available under accession numbers SRR13611586 to SRR13611623.

## Author contributions

HYu wrote the manuscript and analyzed data. WZ wrote the manuscript and conducted field sampling and experimental work. HYi performed research and contributed new methods or models. RZ contributed substantially to manuscript drafting. JW conceived the idea and designed the experiments. All authors contributed to the article and approved the submitted version.

## Funding

This study was supported by National Key R&D Program of China (2019YFA0607100), National Natural Science Foundation of China (42225708 and 91851117), and CAS Key Research Program of Frontier Sciences (QYZDB-SSW-DQC043).

## Conflict of interest

The authors declare that the research was conducted in the absence of any commercial or financial relationships that could be construed as a potential conflict of interest.

## Publisher’s note

All claims expressed in this article are solely those of the authors and do not necessarily represent those of their affiliated organizations, or those of the publisher, the editors and the reviewers. Any product that may be evaluated in this article, or claim that may be made by its manufacturer, is not guaranteed or endorsed by the publisher.

## References

[ref1] Abell R. Thieme M. L. Revenga C. Bryer M. Kottelat M. Bogutskaya N.. (2008). Freshwater ecoregions of the world: a new map of biogeographic units for freshwater biodiversity conservation. Bioscience 58, 403–414. doi: 10.1641/B580507

[ref2] Apprill A. McNally S. Parsons R. Weber L. (2015). Minor revision to V4 region SSU rRNA 806R gene primer greatly increases detection of SAR11 bacterioplankton. Aquat. Microb. Ecol. 75, 129–137. doi: 10.3354/ame01753

[ref3] Aspin T. W. H. Matthews T. J. Khamis K. Milner A. M. Wang Z. O'Callaghan M. J.. (2018). Drought intensification drives turnover of structure and function in stream invertebrate communities. Ecography 41, 1992–2004. doi: 10.1111/ecog.03711

[ref4] Awata T. Tanabe K. Kindaichi T. Ozaki N. Ohashi A. (2012). Influence of temperature and salinity on microbial structure of marine anammox bacteria. Water Sci. Technol. 66, 958–964. doi: 10.2166/wst.2012.234, PMID: 22797222

[ref5] Baselga A. (2010). Partitioning the turnover and nestedness components of beta diversity. Glob. Ecol. Biogeogr. 19, 134–143. doi: 10.1111/j.1466-8238.2009.00490.x

[ref6] Baselga A. Orme C. D. L. (2012). Betapart: an R package for the study of beta diversity. Methods Ecol. Evol. 3, 808–812. doi: 10.1111/j.2041-210X.2012.00224.x

[ref7] Baselga A. Orme D. Villeger S. De Bortoli J. Leprieur F. Baselga M. A. (2018). Package ‘betapart’.

[ref8] Boer W. D. Folman L. B. Summerbell R. C. Boddy L. (2005). Living in a fungal world: impact of fungi on soil bacterial niche development. FEMS Microbiol. Rev. 29, 795–811. doi: 10.1016/j.femsre.2004.11.005, PMID: 16102603

[ref9] Borcard D. Legendre P. (2002). All-scale spatial analysis of ecological data by means of principal coordinates of neighbour matrices. Ecol. Model. 153, 51–68. doi: 10.1016/S0304-3800(01)00501-4

[ref10] Bowen J. L. Crump B. C. Deegan L. A. Hobbie J. E. (2009). Salt marsh sediment bacteria: their distribution and response to external nutrient inputs. ISME J. 3, 924–934. doi: 10.1038/ismej.2009.44, PMID: 19421233

[ref11] Cai Z. Jin T. Li C. Ofterdinger U. Zhang S. Ding A.. (2016). Is China's fifth-largest inland lake to dry-up? Incorporated hydrological and satellite-based methods for forecasting Hulun lake water levels. Adv. Water Resour. 94, 185–199. doi: 10.1016/j.advwatres.2016.05.010

[ref12] Caporaso J. G. Bittinger K. Bushman F. D. DeSantis T. Z. Andersen G. L. Knight R. (2010a). PyNAST: a flexible tool for aligning sequences to a template alignment. Bioinformatics 26, 266–267. doi: 10.1093/bioinformatics/btp636, PMID: 19914921 PMC2804299

[ref13] Caporaso J. G. Kuczynski J. Stombaugh J. Bittinger K. Bushman F. D. Costello E. K.. (2010b). QIIME allows analysis of high-throughput community sequencing data. Nat. Methods 7, 335–336. doi: 10.1038/nmeth.f.303, PMID: 20383131 PMC3156573

[ref14] Cardoso P. Rigal F. Carvalho J. C. Fortelius M. Borges P. A. V. Podani J.. (2014). Partitioning taxon, phylogenetic and functional beta diversity into replacement and richness difference components. J. Biogeogr. 41, 749–761. doi: 10.1111/jbi.12239

[ref15] Castro E. Siqueira T. Melo A. S. Bini L. M. Landeiro V. L. Schneck F. (2019). Compositional uniqueness of diatoms and insects in subtropical streams is weakly correlated with riffle position and environmental uniqueness. Hydrobiologia 842, 219–232. doi: 10.1007/s10750-019-04037-8

[ref16] Chuai X. Chen X. Yang L. Zeng J. Miao A. Zhao H. (2012). Effects of climatic changes and anthropogenic activities on lake eutrophication in different ecoregions. Int. J. Environ. Sci. Technol. 9, 503–514. doi: 10.1007/s13762-012-0066-2

[ref17] Coelho M. S. Carneiro M. A. A. Branco C. A. Borges R. A. X. Fernandes G. W. (2018). Species turnover drives β-diversity patterns across multiple spatial scales of plant-galling interactions in mountaintop grasslands. PLoS One 13:e0195565. doi: 10.1371/journal.pone.0195565, PMID: 29775458 PMC5959069

[ref18] da Silva P. G. Hernández M. I. M. Heino J. (2018). Disentangling the correlates of species and site contributions to beta diversity in dung beetle assemblages. Divers. Distrib. 24, 1674–1686. doi: 10.1111/ddi.12785

[ref19] Dobrovolski R. Melo A. S. Cassemiro F. A. S. Diniz-Filho J. A. F. (2012). Climatic history and dispersal ability explain the relative importance of turnover and nestedness components of beta diversity. Glob. Ecol. Biogeogr. 21, 191–197. doi: 10.1111/j.1466-8238.2011.00671.x

[ref20] Edgar R. C. (2010). Search and clustering orders of magnitude faster than BLAST. Bioinformatics 26, 2460–2461. doi: 10.1093/bioinformatics/btq461, PMID: 20709691

[ref21] Elith J. Leathwick J. R. Hastie T. (2008). A working guide to boosted regression trees. J. Anim. Ecol. 77, 802–813. doi: 10.1111/j.1365-2656.2008.01390.x, PMID: 18397250

[ref22] Encalada A. C. Flecker A. S. Poff N. L. Suárez E. Herrera-R G. A. Ríos-Touma B.. (2019). A global perspective on tropical montane rivers. Science 365, 1124–1129. doi: 10.1126/science.aax1682, PMID: 31515386

[ref23] Epele L. B. Brand C. Miserendino M. L. (2019). Ecological drivers of alpha and beta diversity of freshwater invertebrates in arid and semiarid Patagonia (Argentina). Sci. Total Environ. 678, 62–73. doi: 10.1016/j.scitotenv.2019.04.392, PMID: 31075604

[ref24] Feld C. K. Segurado P. Gutiérrez-Cánovas C. (2016). Analysing the impact of multiple stressors in aquatic biomonitoring data: a ‘cookbook’ with applications in *R*. Sci. Total Environ. 573, 1320–1339. doi: 10.1016/j.scitotenv.2016.06.243, PMID: 27499499

[ref25] Fry E. L. Manning P. Macdonald C. Hasegawa S. de Palma A. Power S. A.. (2016). Shifts in microbial communities do not explain the response of grassland ecosystem function to plant functional composition and rainfall change. Soil Biol. Biochem. 92, 199–210. doi: 10.1016/j.soilbio.2015.10.006

[ref26] Fu C. Wu H. Zhu Z. Song C. Xue B. Wu H.. (2021). Exploring the potential factors on the striking water level variation of the two largest semi-arid-region lakes in northeastern Asia. Catena 198:105037. doi: 10.1016/j.catena.2020.105037

[ref27] Gaston K. J. (2000). Global patterns in biodiversity. Nature 405, 220–227. doi: 10.1038/35012228 10821282

[ref28] González-Trujillo J. D. Donato-Rondon J. C. Muñoz I. Sabater S. (2020a). Historical processes constrain metacommunity structure by shaping different pools of invertebrate taxa within the Orinoco basin. Divers. Distrib. 26, 49–61. doi: 10.1111/ddi.12996

[ref29] González-Trujillo J. D. Pedraza-Garzón E. Donato-Rondon J. C. Sabater S. (2020b). Ecoregional characteristics drive the distribution patterns of Neotropical stream diatoms. J. Phycol. 56, 1053–1065. doi: 10.1111/jpy.13005, PMID: 32320068

[ref30] Goslee S. Urban D. (2007). Ecodist: Dissimilarity-based Functions for Ecological Analysis. R Package Version 1.

[ref31] Gutiérrez-Cánovas C. Millán A. Velasco J. Vaughan I. P. Ormerod S. J. (2013). Contrasting effects of natural and anthropogenic stressors on beta diversity in river organisms. Glob. Ecol. Biogeogr. 22, 796–805. doi: 10.1111/geb.12060

[ref32] Haas B. J. Gevers D. Earl A. M. Feldgarden M. Ward D. V. Giannoukos G.. (2011). Chimeric 16S rRNA sequence formation and detection in sanger and 454-pyrosequenced PCR amplicons. Genome Res. 21, 494–504. doi: 10.1101/gr.112730.110, PMID: 21212162 PMC3044863

[ref33] Harrell F. (2019). Package ‘Hmisc’. R Package Version 4.3-0.

[ref34] Hatosy S. M. Martiny J. B. H. Sachdeva R. Steele J. Fuhrman J. A. Martiny A. C. (2013). Beta diversity of marine bacteria depends on temporal scale. Ecology 94, 1898–1904. doi: 10.1890/12-2125.1, PMID: 24279260

[ref35] Hazzi N. A. Moreno J. S. Ortiz-Movliav C. Palacio R. D. (2018). Biogeographic regions and events of isolation and diversification of the endemic biota of the tropical Andes. Proc. Natl. Acad. Sci. 115, 7985–7990. doi: 10.1073/pnas.1803908115, PMID: 30018064 PMC6077705

[ref36] Hoorn C. Wesselingh F. P. ter Steege H. Bermudez M. A. Mora A. Sevink J.. (2010). Amazonia through time: Andean uplift, climate change, landscape evolution, and biodiversity. Science 330, 927–931. doi: 10.1126/science.1194585, PMID: 21071659

[ref37] Huang X. Chen W. Cai Q. (2000). Survey, observation and analysis of Lake ecology (in Chinese). China Standard Press, Beijing (in Chinese).

[ref38] Ihrmark K. Bödeker I. T. M. Cruz-Martinez K. Friberg H. Kubartova A. Schenck J.. (2012). New primers to amplify the fungal ITS2 region – evaluation by 454-sequencing of artificial and natural communities. FEMS Microbiol. Ecol. 82, 666–677. doi: 10.1111/j.1574-6941.2012.01437.x, PMID: 22738186

[ref39] Janzen D. H. (1967). Why mountain passes are higher in the tropics. Am. Nat. 101, 233–249. doi: 10.1086/282487

[ref40] Landeiro V. L. Franz B. Heino J. Siqueira T. Bini L. M. (2018). Species-poor and low-lying sites are more ecologically unique in a hyperdiverse Amazon region: evidence from multiple taxonomic groups. Divers. Distrib. 24, 966–977. doi: 10.1111/ddi.12734

[ref41] Legendre P. (2014). Interpreting the replacement and richness difference components of beta diversity. Glob. Ecol. Biogeogr. 23, 1324–1334. doi: 10.1111/geb.12207

[ref42] Legendre P. De Cáceres M. (2013). Beta diversity as the variance of community data: dissimilarity coefficients and partitioning. Ecol. Lett. 16, 951–963. doi: 10.1111/ele.12141, PMID: 23809147

[ref43] Li S. Chen J. Xiang J. Pan Y. Huang Z. Wu Y. (2019). Water level changes of Hulun Lake in Inner Mongolia derived from Jason satellite data. J. Vis. Commun. Image Represent. 58, 565–575. doi: 10.1016/j.jvcir.2018.12.031

[ref44] Li C. Ma W. Shi X. Liao W. (2006). Reconstruction of the hydrology series and simulation of salinity in ungauged Lake Hulun. J. Lake Sci. 18, 13–20. doi: 10.18307/2006.0102

[ref45] Liang L. Li C. Shi X. Tian Y. Zhang L. (2016). Analysis on the eutrophication trends and affecting factors in Lake Hulun, 2006-2015. J. Lake Sci. 28, 1265–1273. doi: 10.18307/2016.0612

[ref46] Liaw A. Wiener M. (2002). Classification and regression by random Forest. R News 2, 18–22.

[ref47] Lichstein J. W. (2007). Multiple regression on distance matrices: a multivariate spatial analysis tool. Plant Ecol. 188, 117–131. doi: 10.1007/s11258-006-9126-3

[ref48] Lima M. S. Schneck F. They N. H. Crossetti L. O. Bohnenberger J. E. McMahon K. D.. (2020). Turnover is replaced by nestedness with increasing geographical distance in bacterial communities of coastal shallow lakes. Mar. Freshw. Res. 71, 1086–1098. doi: 10.1071/MF19110

[ref49] Mantel N. (1967). The detection of disease clustering and a generalized regression approach. Cancer Res. 27, 209–220. PMID: 6018555

[ref50] Martiny J. B. H. Eisen J. A. Penn K. Allison S. D. Horner-Devine M. C. (2011). Drivers of bacterial β-diversity depend on spatial scale. Proc. Natl. Acad. Sci. 108, 7850–7854. doi: 10.1073/pnas.1016308108, PMID: 21518859 PMC3093525

[ref51] Medeiros C. R. Hepp L. U. Patrício J. Molozzi J. (2016). Tropical estuarine macrobenthic communities are structured by turnover rather than nestedness. PLoS One 11:e0161082. doi: 10.1371/journal.pone.0161082, PMID: 27584726 PMC5008822

[ref52] Mena J. L. Vázquez-Domínguez E. (2005). Species turnover on elevational gradients in small rodents. Glob. Ecol. Biogeogr. 14, 539–547. doi: 10.1111/j.1466-822X.2005.00189.x

[ref53] Meynard C. N. Devictor V. Mouillot D. Thuiller W. Jiguet F. Mouquet N. (2011). Beyond taxonomic diversity patterns: how do α, β and γ components of bird functional and phylogenetic diversity respond to environmental gradients across France? Glob. Ecol. Biogeogr. 20, 893–903. doi: 10.1111/j.1466-8238.2010.00647.x

[ref54] Moss B. Kosten S. Meerhoff M. Battarbee R. W. Jeppesen E. Mazzeo N.. (2011). Allied attack: climate change and eutrophication. Inland Waters 1, 101–105. doi: 10.5268/IW-1.2.359

[ref55] Paerl H. W. Otten T. G. Kudela R. (2018). Mitigating the expansion of harmful algal blooms across the freshwater-to-marine continuum. Environ. Sci. Technol. 52, 5519–5529. doi: 10.1021/acs.est.7b05950, PMID: 29656639

[ref56] Parada A. E. Needham D. M. Fuhrman J. A. (2016). Every base matters: assessing small subunit rRNA primers for marine microbiomes with mock communities, time series and global field samples. Environ. Microbiol. 18, 1403–1414. doi: 10.1111/1462-2920.13023, PMID: 26271760

[ref57] Qu X. Zhang M. Yang Y. Xie Y. Ren Z. Peng W.. (2019). Taxonomic structure and potential nitrogen metabolism of microbial assemblage in a large hypereutrophic steppe lake. Environ. Sci. Policy 26, 21151–21160. doi: 10.1007/s11356-019-05411-8, PMID: 31119540

[ref58] Rapacciuolo G. Beman J. M. Schiebelhut L. M. Dawson M. N. (2019). Microbes and macro-invertebrates show parallel β-diversity but contrasting α-diversity patterns in a marine natural experiment. Proc. R. Soc. B 286:20190999. doi: 10.1098/rspb.2019.0999, PMID: 31594510 PMC6790787

[ref59] Raposeiro P. M. Saez A. Giralt S. Costa A. C. Gonçalves V. (2018). Causes of spatial distribution of subfossil diatom and chironomid assemblages in surface sediments of a remote deep island lake. Hydrobiologia 815, 141–163. doi: 10.1007/s10750-018-3557-4

[ref60] Rooney R. C. Azeria E. T. (2015). The strength of cross-taxon congruence in species composition varies with the size of regional species pools and the intensity of human disturbance. J. Biogeogr. 42, 439–451. doi: 10.1111/jbi.12400

[ref61] Shade A. Dunn R. R. Blowes S. A. Keil P. Bohannan B. J. M. Herrmann M.. (2018). Macroecology to unite all life, large and small. Trends Ecol. Evol. 33, 731–744. doi: 10.1016/j.tree.2018.08.005, PMID: 30209011

[ref62] Shang Y. Wu X. Wei Q. Dou H. Wang X. Chen J.. (2020). Total arsenic, pH, and sulfate are the Main environmental factors affecting the microbial ecology of the water and sediments in Hulun Lake. China. Front. Microbiol. 11:2258. doi: 10.3389/fmicb.2020.548607 PMC7541820 33072010

[ref63] Si X. Baselga A. Leprieur F. Song X. Ding P. (2016). Selective extinction drives taxonomic and functional alpha and beta diversities in island bird assemblages. J. Anim. Ecol. 85, 409–418. doi: 10.1111/1365-2656.12478, PMID: 26619392

[ref64] Simon M. López-García P. Deschamps P. Restoux G. Bertolino P. Moreira D.. (2016). Resilience of freshwater communities of small microbial eukaryotes undergoing severe drought events. Front. Microbiol. 7:812. doi: 10.3389/fmicb.2016.00812, PMID: 27303393 PMC4885337

[ref65] Socolar J. B. Gilroy J. J. Kunin W. E. Edwards D. P. (2016). How should Beta-diversity inform biodiversity conservation? Trends Ecol. Evol. 31, 67–80. doi: 10.1016/j.tree.2015.11.005, PMID: 26701706

[ref66] Soininen J. Heino J. Wang J. (2018). A meta-analysis of nestedness and turnover components of beta diversity across organisms and ecosystems. Glob. Ecol. Biogeogr. 27, 96–109. doi: 10.1111/geb.12660

[ref67] Sparks D. L. Page A. L. Helmke P. A. Loeppert R. H. (1996). Methods of soil analysis, part 3: Chemical methods 14.

[ref68] Stegen J. C. Lin X. Fredrickson J. K. Chen X. Kennedy D. W. Murray C. J.. (2013). Quantifying community assembly processes and identifying features that impose them. ISME J. 7, 2069–2079. doi: 10.1038/ismej.2013.93, PMID: 23739053 PMC3806266

[ref69] Szabó B. Lengyel E. Padisák J. Stenger-Kovács C. (2019). Benthic diatom metacommunity across small freshwater lakes: driving mechanisms, β-diversity and ecological uniqueness. Hydrobiologia 828, 183–198. doi: 10.1007/s10750-018-3811-9

[ref70] Tranvik L. J. Downing J. A. Cotner J. B. Loiselle S. A. Striegl R. G. Ballatore T. J.. (2009). Lakes and reservoirs as regulators of carbon cycling and climate. Limnol. Oceanogr. 54, 2298–2314. doi: 10.4319/lo.2009.54.6_part_2.2298

[ref71] Walter J. M. Lopes F. A. Lopes-Ferreira M. Vidal L. M. Leomil L. Melo F.. (2018). Occurrence of harmful cyanobacteria in drinking water from a severely drought-impacted semi-arid region. Front. Microbiol. 9:176. doi: 10.3389/fmicb.2018.00176, PMID: 29541063 PMC5835534

[ref72] Wang J. Hu A. Meng F. Zhao W. Yang Y. Soininen J.. (2022). Embracing mountain microbiome and ecosystem functions under global change. New Phytol. 234, 1987–2002. doi: 10.1111/nph.18051, PMID: 35211983

[ref73] Wang J. Legendre P. Soininen J. Yeh C. F. Graham E. Stegen J.. (2020). Temperature drives local contributions to beta diversity in mountain streams: stochastic and deterministic processes. Glob. Ecol. Biogeogr. 29, 420–432. doi: 10.1111/geb.13035

[ref74] Wang X.-B. Lü X.-T. Yao J. Wang Z. W. Deng Y. Cheng W. X.. (2017). Habitat-specific patterns and drivers of bacterial β-diversity in China’s drylands. ISME J. 11, 1345–1358. doi: 10.1038/ismej.2017.11, PMID: 28282041 PMC5437346

[ref75] Wang J. Pan F. Soininen J. Heino J. Shen J. (2016). Nutrient enrichment modifies temperature-biodiversity relationships in large-scale field experiments. Nat. Commun. 7:13960. doi: 10.1038/ncomms13960, PMID: 28000677 PMC5187590

[ref76] Wang J. Shen J. Wu Y. Tu C. Soininen J. Stegen J. C.. (2013). Phylogenetic beta diversity in bacterial assemblages across ecosystems: deterministic versus stochastic processes. ISME J. 7, 1310–1321. doi: 10.1038/ismej.2013.30, PMID: 23446837 PMC3695296

[ref77] Wang X. Van Nostrand J. D. Deng Y. Lü X. Wang C. Zhou J.. (2015). Scale-dependent effects of climate and geographic distance on bacterial diversity patterns across northern China's grasslands. FEMS Microbiol. Ecol. 91:fiv133. doi: 10.1093/femsec/fiv133 26519142

[ref78] Wang S. Wang X. Han X. Deng Y. (2018). Higher precipitation strengthens the microbial interactions in semi-arid grassland soils. Glob. Ecol. Biogeogr. 27, 570–580. doi: 10.1111/geb.12718

[ref79] Wang G. Wang Y. Li Y. Cheng H. (2007). Influences of alpine ecosystem responses to climatic change on soil properties on the Qinghai–Tibet plateau, China. Catena 70, 506–514. doi: 10.1016/j.catena.2007.01.001

[ref80] Wang Q. Yang X. Hamilton B. P. Zhang E. (2012). Linking spatial distributions of sediment diatom assemblages with hydrological depth profiles in a plateau deep-water lake system of subtropical China. Fottea 12, 59–73. doi: 10.5507/fot.2012.005

[ref81] Wang J. Zhang T. Li L. Li J. Feng Y. Lu Q. (2017). The patterns and drivers of bacterial and fungal β-diversity in a typical dryland ecosystem of Northwest China. Front. Microbiol. 8:2126. doi: 10.3389/fmicb.2017.02126, PMID: 29176963 PMC5686094

[ref82] Wen R. Xiao J. Chang Z. Zhai D. Xu Q. Li Y.. (2010). Holocene climate changes in the mid-high-latitude-monsoon margin reflected by the pollen record from Hulun Lake, northeastern Inner Mongolia. Quatern. Res. 73, 293–303. doi: 10.1016/j.yqres.2009.10.006

[ref83] Whittaker R. H. (1960). Vegetation of the Siskiyou Mountains, Oregon and California. Ecol. Monogr. 30, 279–338. doi: 10.2307/1943563

[ref84] Woolway R. I. Kraemer B. M. Lenters J. D. Merchant C. J. O’Reilly C. M. Sharma S. (2020). Global lake responses to climate change. Nat. Rev. Earth Environ. 1, 388–403. doi: 10.1038/s43017-020-0067-5

[ref85] Wu K. Zhao W. Li M. Picazo F. Soininen J. Shen J.. (2020). Taxonomic dependency of beta diversity components in benthic communities of bacteria, diatoms and chironomids along a water-depth gradient. Sci. Total Environ. 741:140462. doi: 10.1016/j.scitotenv.2020.140462, PMID: 32886961

[ref86] Xie Z. He J. Lü C. Zhang R. Zhou B. Mao H.. (2015). Organic carbon fractions and estimation of organic carbon storage in the lake sediments in Inner Mongolia plateau, China. Environ. Earth Sci. 73, 2169–2178. doi: 10.1007/s12665-014-3568-z

[ref87] Yamaoka K. Nakagawa T. Uno T. (1978). Application of Akaike's information criterion (AIC) in the evaluation of linear pharmacokinetic equations. J. Pharmcokinet. Biopharm. 6, 165–175. doi: 10.1007/BF01117450, PMID: 671222

[ref88] Yeh C.-F. Soininen J. Teittinen A. Wang J. (2019). Elevational patterns and hierarchical determinants of biodiversity across microbial taxonomic scales. Mol. Ecol. 28, 86–99. doi: 10.1111/mec.14935, PMID: 30427089

[ref89] Yuan H. Meng F. Yamamoto M. Liu X. Dong H. Shen J.. (2021). Linking historical vegetation to bacterial succession under the contrasting climates of the Tibetan plateau. Ecol. Indic. 126:107625. doi: 10.1016/j.ecolind.2021.107625

[ref90] Zhai D. Xiao J. Zhou L. Wen R. Chang Z. Wang X.. (2011). Holocene east Asian monsoon variation inferred from species assemblage and shell chemistry of the ostracodes from Hulun Lake, Inner Mongolia. Quatern. Res. 75, 512–522. doi: 10.1016/j.yqres.2011.02.008

[ref91] Zhang W. Chen R. Meng F. Yuan H. Geng M. Cheng L.. (2021). Ecosystem functioning is linked to microbial evenness and community composition along depth gradient in a semiarid lake. Ecol. Indic. 132:108314. doi: 10.1016/j.ecolind.2021.108314

[ref92] Zhou J. Deng Y. Shen L. Wen C. Yan Q. Ning D.. (2016). Temperature mediates continental-scale diversity of microbes in forest soils. Nat. Commun. 7:12083. doi: 10.1038/ncomms12083, PMID: 27377774 PMC4935970

[ref93] Zinger L. Lejon D. P. Baptist F. Bouasria A. Aubert S. Geremia R. A.. (2011). Contrasting diversity patterns of crenarchaeal, bacterial and fungal soil communities in an alpine landscape. PLoS One 6:e19950. doi: 10.1371/journal.pone.0019950, PMID: 21589876 PMC3093402

