# Supplementary material for: Taxonomic dependency of beta diversity for bacteria, archaea, and fungi in a semi-arid lake
Source: Front Microbiol. 2022 Nov 3;13:998496. doi: 10.3389/fmicb.2022.998496 (PMC9670189; doi:10.3389/fmicb.2022.998496)
Supplement: Supplementary file 2 [file Data_Sheet_2.pdf]

Supplementary Material

Table S1 The list of predictors used in this study.

Predictors	Variables	Acronym	Value range	Units
Water factors	Depth	water_Depth	3.8-6.4	m
	Temperature	water_Temp	16.2-19.5	°C
	Dissolved oxygen	water_DO	101.7-118.3	mg/L
	pH	water_pH	9.09-9.16	
	Electrical conductivity	water_EC	-16 to +54.6	µs/cm
	Secchi depth	water_SD	0.29-0.38	m
	NO_3^--N	water_NO3.N	0-0.0079	µg/mL
	NO_2^--N	water_NO2.N	0-0.0012	µg/mL
	NH_4^+-N	water_NH4.N	0.013-0.047	mg/L
	Total nitrogen	water_TN	0.806-1.351	mg/L
	Total phosphorus	water_TP	0.073-0.167	mg/L
	PO_4^{3-}-P	water_PO4.P	0.038-0.093	mg/L
Sediment factors	pH	sedi_pH	8.5-8.8	
	Electrical conductivity	sedi_EC	126.8-1021	µs/cm
	Water content	sedi_WC	16.122-85.314	%
	Grain size*	sedi_GS	5.826-98.939	µm
	Dissolved organic carbon	sedi_DOC	0.462-2.279	mg/kg
	Total carbon	sedi_TC	2320-56800	mg/kg
	NO_2^--N	sedi_NO2.N	0.036-0.364	mg/kg
	NO_3^--N	sedi_NOX.N	0.009-0.034	µg/kg
	NH_4^+-N	sedi_NH4.N	0.005-0.024	µg/kg
	Total nitrogen	sedi_TN	204-3400	mg/kg
	PO_4^{3-}-P	sedi_PO4.P	2.381-13.513	mg/kg
	Total phosphorus	sedi_TP	179-1100	mg/kg

*Grain size: the grain size is less than 32 µm.

Table S2 Summary of the linear or quadratic models evaluated for the water-depth patterns regarding LCBD and total beta diversity and their components. Pearson correlation was performed to identify the relationships between LCBD (including $LCBD_{Repl}$ and $LCBD_{Nes}$) and the water depth, and Mantel test was applied to examine the water-depth patterns of the total beta diversity and its two components. Depth: water depth. R^2: the proportion of explained variance.

	**Microbes**	**Depth**	**R^2**	**P-value**	**slope**
LCBD	Bacteria	Depth	0.3564	0.0041	-0.0030
	Archaea	Depth	0.2323	0.0211	-0.0043
	Fungi	Depth	-0.0075	0.3652	0.0031
$LCBD_{Repl}$	Bacteria	Depth	0.1429	0.0617	-0.0029
	Archaea	Depth	0.3114	0.0077	-0.0050
	Fungi	Depth	-0.0308	0.5061	0.0011
$LCBD_{Nes}$	Bacteria	Depth	-0.0541	0.7866	-0.0037
	Archaea	Depth	-0.0413	0.6003	0.0083
	Fungi	Depth	-0.0327	0.5029	0.0105
Total beta	Bacteria	Depth	0.29197	1.441711e-14	0.0253
	Archaea	Depth	0.15393	6.661374e-08	0.0414
	Fungi	Depth	0.00313	2.170750e-01	-0.0138
Turnover	Bacteria	Depth	0.23478	1.130916e-11	0.0282
	Archaea	Depth	0.16308	2.590721e-08	0.0451
	Fungi	Depth	-0.00152	3.904263e-01	-0.0050
Nestedness	Bacteria	Depth	-0.00111	3.694045e-01	-0.0029
	Archaea	Depth	0.00295	2.220016e-01	-0.0037
	Fungi	Depth	-0.00282	4.712695e-01	-0.0089

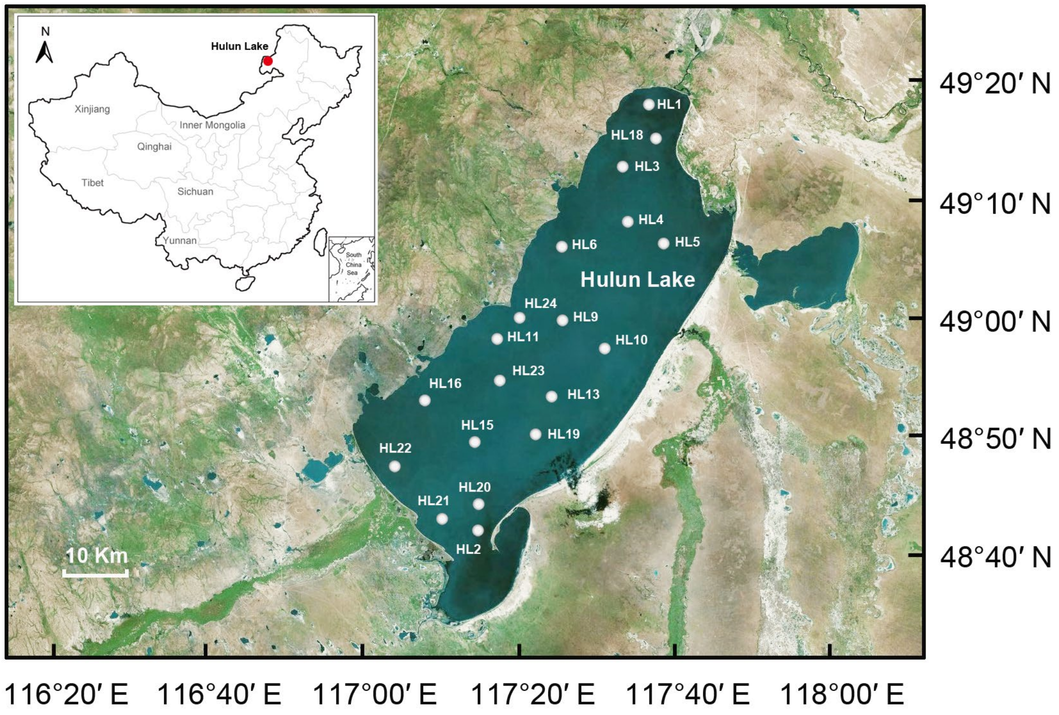

Figure S1 Sampling map of Hulun Lake.

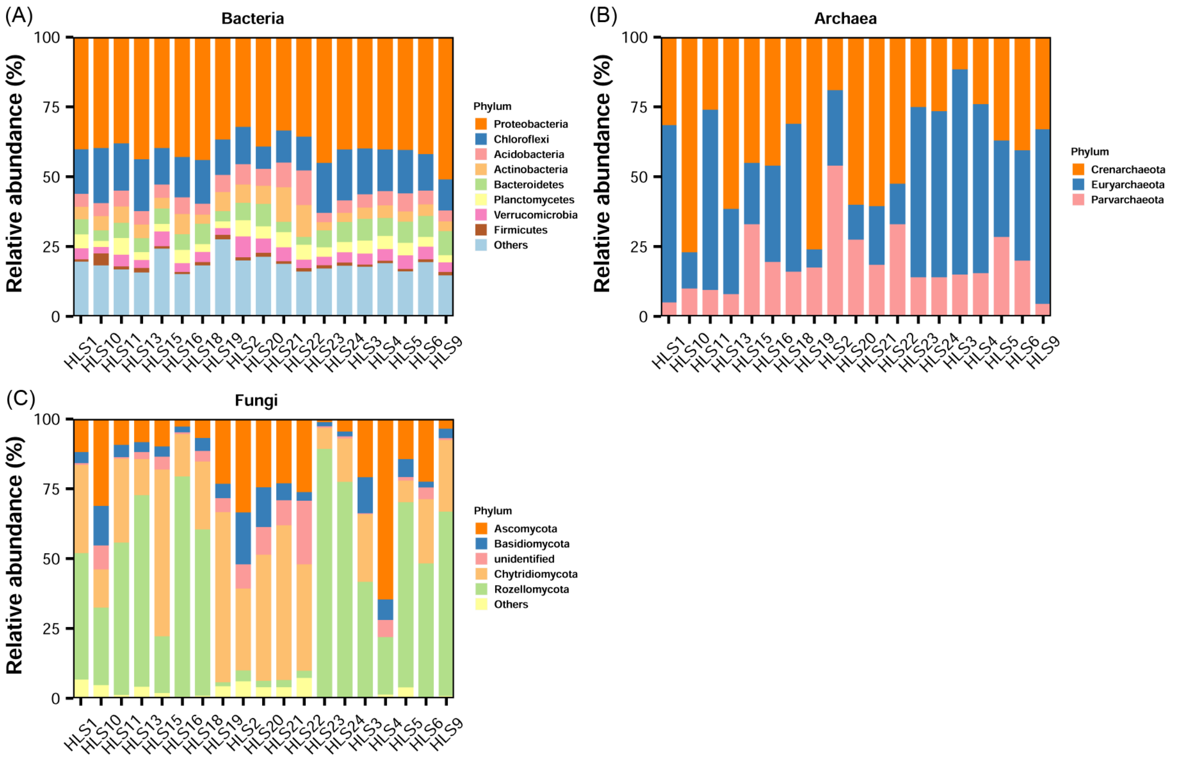

Figure S2 The dominant bacterial, archaeal and fungal phyla in the sediments of Hulun Lake.

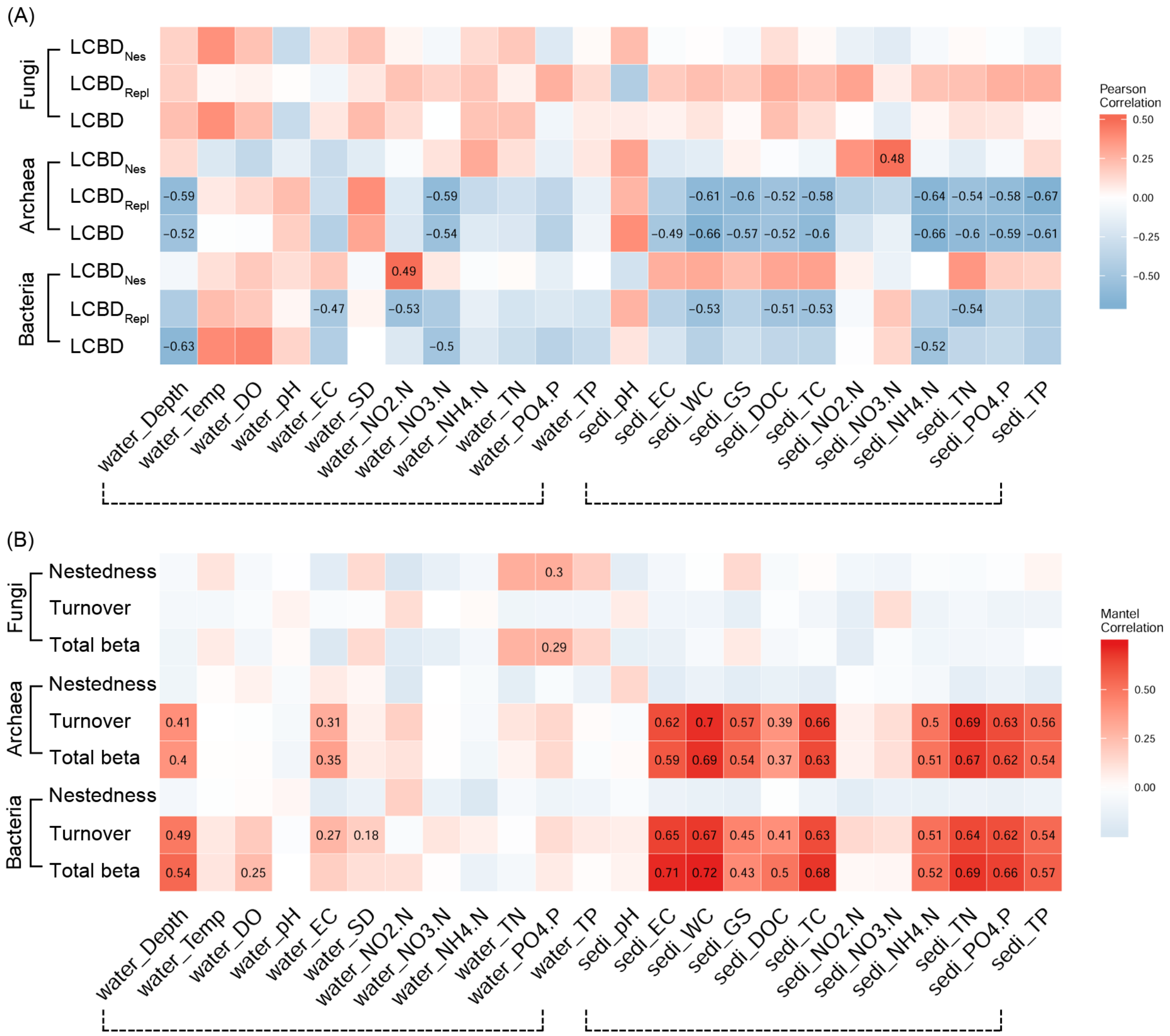

Figure S3 Correlations between environmental variables and the LCBD (A), total beta diversity (B) or their components. Pearson correlation (A) was performed to identify the relationships between environment variables (including water and sediment factors) and the LCBD (including $LCBD_{Repl}$ and $LCBD_{Nes}$), and Mantel test (B) was applied to examine the relationships between environment variables and the total beta diversity or its two components. The value indicates the significant (P < 0.05) correlation. The abbreviations of these variables are listed in Table S1.

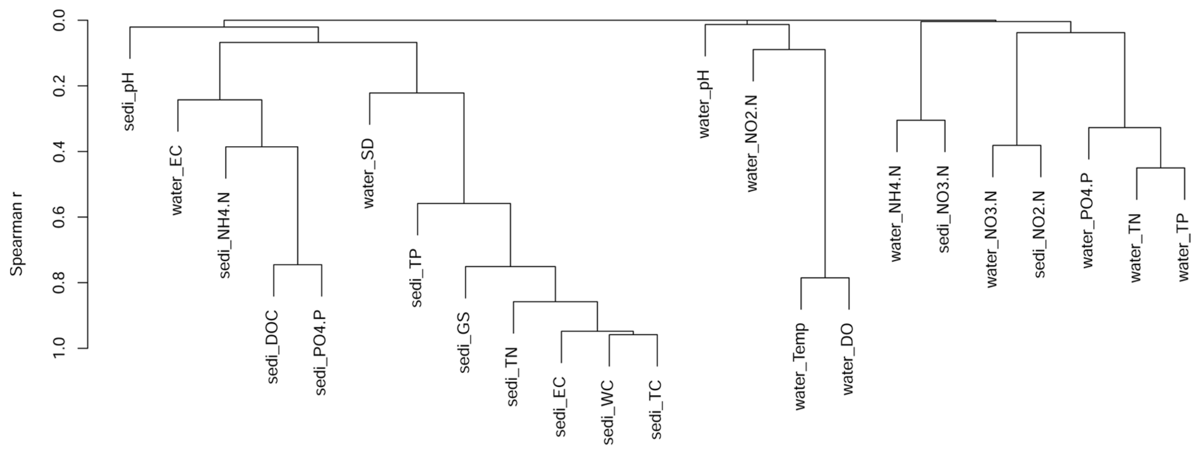

Figure S4 Spearman correlation between the environmental variables. To avoid the effects of multi-collinearity, the redundancy of the environmental factors is evaluated using the function 'varclus' of package Hmisc. Only one variable is retained when a high correlation (Spearman $r > 0.7$) exists between these variables, hence, sedi_DOC, sedi_TN and water_DO are retained. The abbreviations of these variables are listed in Table S1.
